# Design of a multi-walled carbon nanotube field emitter with micro vacuum gauge

**DOI:** 10.1186/1556-276X-8-143

**Published:** 2013-03-28

**Authors:** Ki-Young Dong, Yang Doo Lee, Byung Hyun Kang, Jinnil Choi, Byeong-Kwon Ju

**Affiliations:** 1 Display and Nanosystem Laboratory, College of Engineering, Korea University, Anam-dong, Seongbuk-gu, Seoul, 136-713, Republic of Korea; 2 Department of Mechanical Engineering, Hanbat National University, Daejeon, 305-719, Republic of Korea

**Keywords:** Multi-walled carbon nanotubes, Field emitter, Vacuum gauge, Outgassing

## Abstract

The variation of vacuum level inside a field emission device when electron is emitted from multi-walled carbon nanotubes (MWCNTs) by electric field was measured where MWCNT gauge packaged with a vacuum device was used to measure the degree of a vacuum until the end of the vacuum device life. It was found that the electrical properties of MWCNTs altered with the degree of a vacuum. We fabricated MWCNT gauge which were printed and pasted by the screen printer. In this paper, we report the successful detection of the ionization of gases in vacuum state.

## Background

Carbon nanotube (CNT) is one of the most promising materials for a field emitter due to its remarkable electrical conductivity, chemical and mechanical stability, and characteristics having unique structures such as high aspect ratio [1-5]. Many researches have been highly devoted to developing a practical application for the commercialization of field emitter, but there are still some problems to be solved such as the lifetime of the emitter [6-10]. There are many factors that affect the emitter lifetime working in a state of vacuum. Among them, outgassing generated during emission is inarguably one of the most critical factors [11-13]. Especially, some organic binders can still remain after firing when the multi-walled carbon nanotube (MWCNT) emitter is made in paste and be the source to release gas in the vacuum panel. The outgassing can give a severe damage to the vacuum microelectronic device by electrical arcing and ion bombardment onto a cathode or an anode. In addition to the physical damages, some gases can cause chemical etching to the MWCNT emitter. These highlight that controlling the outgassing is a key issue for emission devices prepared from the paste.

Therefore, it is very important to monitor the vacuum level in a vacuum device in order to maintain satisfying field emission properties. To measure the inner vacuum of the device, the vacuum gauge should be integrated to the vacuum device without affecting the device. MWCNTs were used to fabricate the real time-monitoring vacuum gauge that satisfies these conditions. MWCNTs facilitate the fabrication of a microstructure and this microstructure was used to build the micro vacuum gauge that could be set up in the device. Here, we demonstrate a simple screen-printed MWCNT device that combines the MWCNT field emission and MWCNT-based vacuum gauge for the measurement of the vacuum level. Also, the MWCNT vacuum gauge packaged with a vacuum device is used to measure the lifetime of the vacuum device.

## Methods

The weight ratio of MWCNT/glass frit/indium tin oxide (ITO) powder/Ethyl cellulose/*α*-terpineol was 1:10:2:9:100. MWCNT powder grown by chemical vapor deposition was used as an electron emission source and glass frit as an inorganic binder to enhance the adhesion between MWCNT and the substrate after firing. MWCNT field emitters and the vacuum gauge were fabricated by the screen-printing process, where the field emitters were used as electron source. In the mixture of MWCNTs, the organic binder was premixed through an ultra-sonication for 30 min. Then, a three-roll milling process was carried out for mixing and dispersion of MWCNTs in the organic binder to form a polymer matrix. Mechanically well-dispersed MWCNT paste was printed onto an ITO glass. The residue of organic binder leads to problems such as outgassing and arcing during a field emission measurement. Therefore, organic materials in paste were removed by drying the printed MWCNT paste in the furnace for 30 min at 400°C to obtain stable emission characteristics.

The gas sensing and field emission areas were printed in cathode plate. The MWCNT paste film was fired at 350°C in nitrogen (N_2_) ambient in a furnace. Finally, the MWCNTs in printed cathode layer are randomly distributed in a matrix material. Therefore, their emission characteristics are poor compared to, for instance, highly ordered arrays of vertically aligned MWCNTs. The surface treatment of printed MWCNTs was performed for vertical alignment as well as protrusion of MWCNTs from the surface to increase of field emission current and to improve the sensitivity of the vacuum gauge.

**Figure 1:**
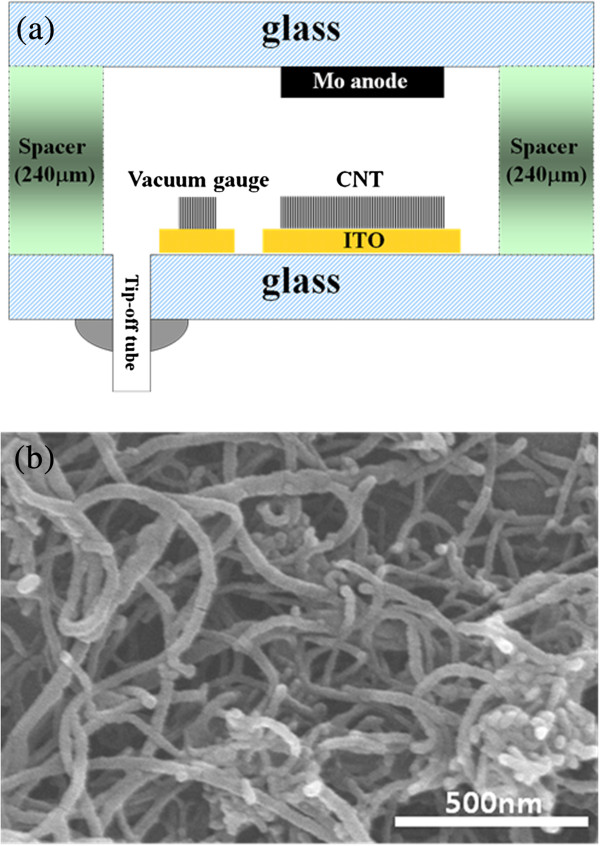
**Structure of MWCNT device and FE-SEM image of MWCNT paste after heat treatment.** (**a**) Structure of the MWCNT device. (**b**) FE-SEM image of MWCNT paste printed on ITO glass substrate after heat treatment.

**Figure 2:**
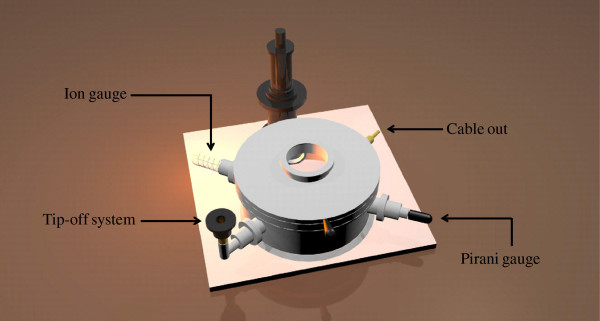
Schematic of the high vacuum chamber with tip-off system.

The proposed vacuum device is a vacuum gauge with a field emitter structure, as shown in Figure 1. The MWCNT vacuum gauge area was connected with a pair of ITO electrodes on the glass plate of cathode to measure the electrical parameters. In addition, the molybdenum (Mo) patterned on glass was used as the anode plate. Two glass plates (cathode and anode glasses) were assembled by a distance of 240 μm. When the cathode plate was applied with high voltage, field emission current was obtained. Figure 2 shows the schematic of the high vacuum chamber. To test the performance of the field emission and measurement of current level, during the experiment, the two MWCNT vacuum devices, a high vacuum chamber, and the tip-off system were connected to the same vacuum level. MWCNT for the vacuum gauge was packaged by tip-off through a vacuum system at a pressure of 1.3 × 10^-6^ Torr. The vacuum gauge output was measured by using a source meter (Keithley 2400, Cleveland, OH, USA) and LabVIEW software (National Instruments Corp., Austin, TX, USA).

## Results and discussion

**Figure 3:**
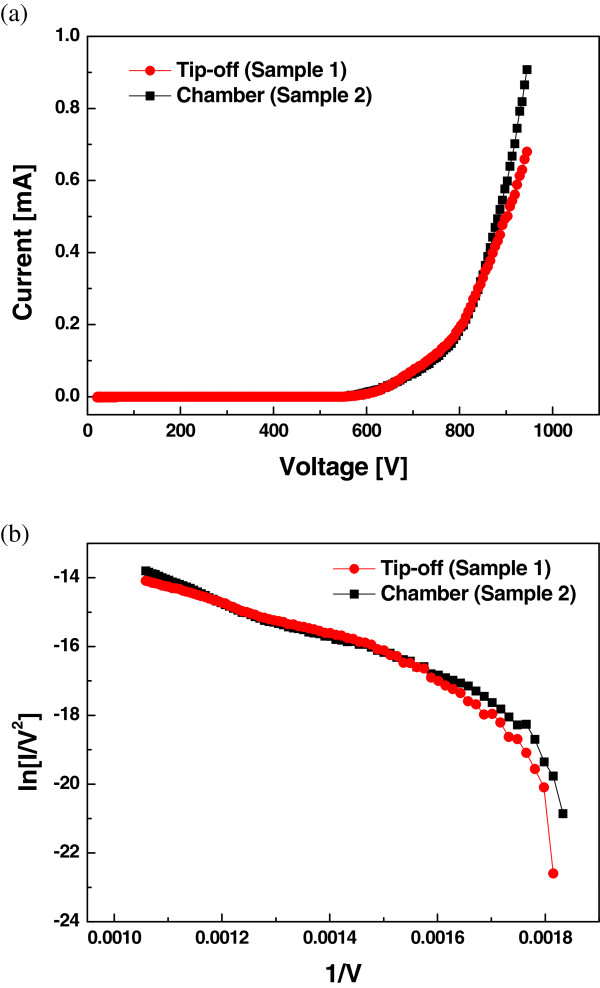
**Current versus voltage properties for the printed MWCNT paste film (a).** The F-N plots (**b**).

Figure 3a shows the field emission characteristic of printed CNT before and after vacuum packaging. The turn-on field required to reach a current density of 10 μA/cm^2^ was 2.54 V/μm (610 V) and 2.5 V/μm (600 V) with tip-off (Sample 1) and vacuum chamber (Sample 2) processes, respectively. Figure 3b shows the Fowler-Nordheim (F-N) plot (*ln(I/V*^*2*^*)* versus *1/V*) and nonlinear slopes. At an applied voltage of 950V, the emission current of MWCNT film decreased from 0.9 to 0.7 mA after the tip-off. The reasons for this could be explained by vacuum level change due to outgassing inside the flat panel during tip-off process.

**Figure 4:**
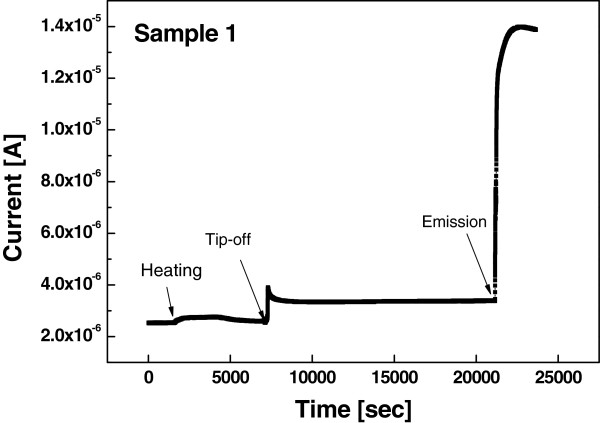
Current changes of the MWCNT device during tip-off process.

Figure 4 exhibits the plot of the current versus time of the packaged device which was loaded in the vacuum chamber tip-off system (Sample 1). In this experiment, applied voltage to the vacuum gauge was 1 V. The measurement of the current was initiated after saturation was reached by the rotary pump and the turbo pump. As the gauge was heated by the tip-off heater from 2,000 to 2,300 s, the current increased after heater was turned on and decreased gradually following the turning-off of the heater. This phenomenon can be probably explained by the fact that there is limit in the amount of outgas that can be removed by the pumps. When the vacuum status approached to 1.2 × 10^-6^ Torr, the device was tipped off. The tip-off process was as follows: glass tip was located on the heater, which was in the vacuum chamber, and heated. The heater made the temperature exceed the melting point of the glass in a few minutes. At this instance, melted glass was held together for a short time to close the glass tip and separated from the vacuum pump. The outgas generated by heating and field emission resulted in the increase of the current, i.e., the current increased upon exposure to field emission outgases.

**Figure 5:**
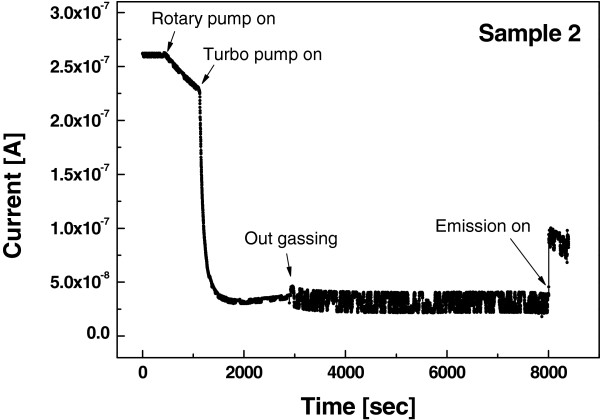
Variation of device current in the sequential step of field emission experiment inside high vacuum chamber.

Figure 5 shows the current of the MWCNT vacuum gauge at the device versus time inside high vacuum chamber (Sample 2). From Figure 4, it was found that vacuum level was changed when heat was generated at the tip-off by using the vacuum gauge. Therefore, we measured the change of the current as vacuum level was changed without tip-off, and the device was sealed for more precise measurement. Pirani gauge, a low-level vacuum gauge, provided that the current was decreased at 450 s when the rotary pump was turned on. After the turbo pump was turned on, significant change in the current was observed. After 2,900 s, the vacuum level approached 9.8 × 10^-7^ Torr, and outgassing occurred in the chamber. It seemed that the device current changed because these gases resulted from outgassing adsorbed onto the MWCNTs. The vacuum level was changed from 9.8 × 10^-7^ to 2.8 × 10^-5^ Torr after emission. The current of the vacuum gauge was increased when exposed to field emission outgases.

**Figure 6:**
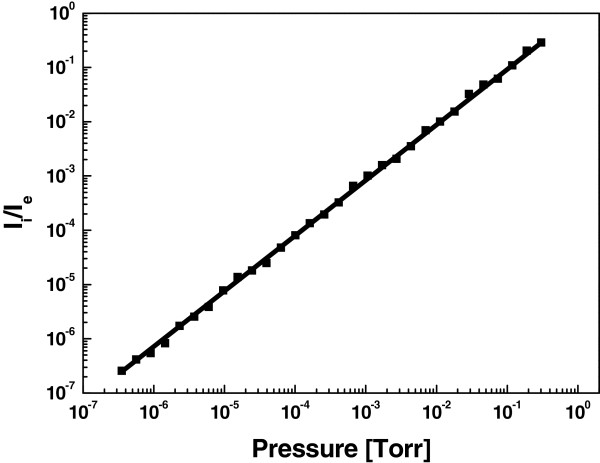
Normalized ion current versus chamber pressure for air.

The sensitivity *K* of the ion gauge can be represented by *K* = *I*_i_*/I*_e_*P, w*here *I*_i_ is the ion current, *I*_e_ is emission current, and *P* is the pressure. The anode voltage and the collector voltage were biased to 800 V and -10 V, respectively. As shown in Figure 6, the gauge showed excellent measurement linearity between normalized ion current (*I*_i_*/I*_e_) and vacuum pressure for air. It can be seen that the ratio of the ion current to the emission current is linear with respect to the air pressure in the range of 10^-7^ to 1 Torr. The sensitivity derived from linear fits of the data was calculated to be approximately 2 Torr^-1^, which is smaller than that of the commercial Bayard-Alpert gauge (BAG) in the range of 8 to 45 Torr^-1^. The gauge sensitivity is dependent on the structure of the vacuum sensor and electrical potential (typical value of 150 to 200 V). The sensitivity of the MWCNT-emitter vacuum gauge was lower compared to the BAG due to short electron paths and higher anode voltage (800 V).

## Conclusions

In this work, the change in inner vacuum of the vacuum-packaged emitter device and the current of printed MWCNT ionization vacuum gauge by field emission were explored. The MWCNT emitter showed excellent emission characteristics under vacuum pressure below 10^-6^ Torr. The MWCNT source vacuum gauge presented good measurement linearity from 10^-7^ to 1 Torr for air. This MWCNT-based gauge is expected to find several applications such as ultrahigh vacuum systems, vacuum inside sealed devices, and field emission devices.

## Competing interests

The authors declare that they have no competing interests.

## Authors’ contributions

The work presented here was carried out in collaboration among all authors. KYD, JC, and BKJ defined the research theme. KYD, JC, and YDL designed the methods and experiments, carried out the laboratory experiments, analyzed the data, interpreted the results, and wrote the paper. BHK and YDL worked on the associated data collection and their interpretation and wrote the paper. KYD, JC, and BKJ designed the experiments, discussed the analyses, and wrote the paper. All authors read and approved the final manuscript.
